# National Outbreak of Multidrug Resistant *Salmonella* Heidelberg Infections Linked to a Single Poultry Company

**DOI:** 10.1371/journal.pone.0162369

**Published:** 2016-09-15

**Authors:** Laura Gieraltowski, Jeffrey Higa, Vi Peralta, Alice Green, Colin Schwensohn, Hilary Rosen, Tanya Libby, Bonnie Kissler, Nicola Marsden-Haug, Hillary Booth, Akiko Kimura, Julian Grass, Amelia Bicknese, Beth Tolar, Stephanie Defibaugh-Chávez, Ian Williams, Matthew Wise, Salmonella Heidelberg Investigation Team

**Affiliations:** 1 Division of Foodborne, Waterborne and Environmental Diseases, Centers for Disease Control and Prevention, Atlanta, Georgia, United States of America; 2 California Department of Public Health, Gardena, Richmond, and Sacramento, California, United States of America; 3 California Emerging Infections Program, Oakland, California, United States of America; 4 Washington State Department of Health, Olympia, Washington, United States of America; 5 Oregon Public Health Division, Portland, Oregon, United States of America; 6 Office of Public Health Science, Food Safety and Inspection Service, United States Department of Agriculture, Washington, DC, United States of America; Health Protection Agency, UNITED KINGDOM

## Abstract

**Importance:**

This large outbreak of foodborne salmonellosis demonstrated the complexity of investigating outbreaks linked to poultry products. The outbreak also highlighted the importance of efforts to strengthen food safety policies related to *Salmonella* in chicken parts and has implications for future changes within the poultry industry.

**Objective:**

To investigate a large multistate outbreak of multidrug resistant *Salmonella* Heidelberg infections.

**Design:**

Epidemiologic and laboratory investigations of patients infected with the outbreak strains of *Salmonella* Heidelberg and traceback of possible food exposures.

**Setting:**

United States. Outbreak period was March 1, 2013 through July 11, 2014

**Patients:**

A case was defined as illness in a person infected with a laboratory-confirmed *Salmonella* Heidelberg with 1 of 7 outbreak pulsed-field gel electrophoresis (PFGE) *Xba*I patterns with illness onset from March 1, 2013 through July 11, 2014. A total of 634 case-patients were identified through passive surveillance; 200/528 (38%) were hospitalized, none died.

**Results:**

Interviews were conducted with 435 case-patients: 371 (85%) reported eating any chicken in the 7 days before becoming ill. Of 273 case-patients interviewed with a focused questionnaire, 201 (74%) reported eating chicken prepared at home. Among case-patients with available brand information, 152 (87%) of 175 patients reported consuming Company A brand chicken. Antimicrobial susceptibility testing was completed on 69 clinical isolates collected from case-patients; 67% were drug resistant, including 24 isolates (35%) that were multidrug resistant. The source of Company A brand chicken consumed by case-patients was traced back to 3 California production establishments from which 6 of 7 outbreak strains were isolated.

**Conclusions:**

Epidemiologic, laboratory, traceback, and environmental investigations conducted by local, state, and federal public health and regulatory officials indicated that consumption of Company A chicken was the cause of this outbreak. The outbreak involved multiple PFGE patterns, a variety of chicken products, and 3 production establishments, suggesting a reservoir for contamination upstream from the production establishments. Sources of bacteria and genes responsible for resistance, such as farms providing birds for slaughter or environmental reservoir on farms that raise chickens, might explain how multiple PFGE patterns were linked to chicken from 3 separate production establishments and many different poultry products.

## Introduction

Nontyphoidal *Salmonella* is the most common bacterial cause of foodborne illness in the United States (US), causing an estimated 1 million illnesses and 400 deaths each year [1]. In 2013, *Salmonella enterica* serotype Heidelberg was the ninth most common serotype among human infections and the third most common serotype among *Salmonella* isolated from retail chicken samples [2–4].

Multistate outbreaks of foodborne bacterial infections in the US are typically identified through PulseNet, a national molecular surveillance network, coordinated by the US Centers for Disease Control and Prevention (CDC). This system relies on clinical laboratories submitting enteric bacteria isolates from ill persons to public health laboratories for subtyping using pulsed-field gel electrophoresis (PFGE). These laboratories upload PFGE patterns to a national database at CDC, where local, state, and federal microbiologists and epidemiologists monitor for clusters of illnesses caused by bacteria with the same PFGE pattern. CDC identifies and monitors at least 200 clusters of *Salmonella* and *E*. *coli* illnesses each year and coordinates multistate epidemiologic investigations to identify common sources of infection, working with regulatory, industry, and other partners to stop outbreaks.

On June 17, 2013, PulseNet identified a cluster of 13 *Salmonella* Heidelberg illnesses with a rare PFGE pattern (*Xba*I pattern JF6X01.0258) reported from California and Washington. During the same time period, *Salmonella* Heidelberg with pattern JF6X01.0258 was also isolated from a chicken breast collected from a California retail store as part of the National Antimicrobial Resistance Monitoring System (NARMS) Retail Meat Surveillance Program. NARMS is a collaboration of the US Food and Drug Administration (FDA), US Department of Agriculture (USDA), state health and agriculture departments, and CDC that monitors antimicrobial resistance in *Salmonella* and other enteric bacteria isolated from, raw meat and poultry, food-producing animals and ill persons. Based on this information, CDC initiated an investigation to determine whether these illnesses were linked to a common source.

## Methods

Human subject protection officers of the National Center for Emerging and Zoonotic Infectious Diseases within the Centers for Disease Control and Prevention determined that these investigations did not meet the definition of research as provided by 45 CFR4 6.102(d) and therefore IRB oversight was not required. The basis for this determination was that the primary purpose of this activity was to identify, characterize, and control disease in response to an immediate public health threat. The purpose of the investigation was explained to all participants and participation was voluntary.

### Outbreak identification and case finding

The initial case definition was a laboratory-confirmed *Salmonella* Heidelberg infection reported to PulseNet with PFGE *Xba*I pattern JF6X01.0258 and illness onset on or after March 1, 2013. During July and August 2013, PulseNet detected 6 additional PFGE clusters of *Salmonella* Heidelberg illnesses that appeared to be related to the ongoing investigation based on the distribution and timing of infections, relatedness of the PFGE patterns, and food histories reported. Because of these similarities, the 7 PFGE clusters were combined into a single investigation. The final case definition was a laboratory-confirmed *Salmonella* Heidelberg infection reported to PulseNet with illness onset from March 1, 2013 through July 11, 2014 and with 1 of 7 PFGE *Xba*I patterns (the outbreak strains): JF6X01.0022 (California only), JF6X01.0041, JF6X01.0045, JF6X01.0122, JF6X01.0258, JF6X01.0326, or JF6X01.0672. Because JF6X01.0022 is the most common *Salmonella* Heidelberg pattern reported nationally and the increase in that pattern was focused in California, only the pattern JF6X01.0022 isolates from California case-patients were included in order to minimize illnesses that were likely unrelated to the outbreak.

### Epidemiologic investigation and statistical analysis

To identify possible sources of infection, state and local public health officials interviewed case-patients about food and environmental exposures occurring in the week before illness onset. Based on initial reported poultry exposures and isolation of outbreak strains from samples of chicken purchased at retail venues, a focused questionnaire was developed that included detailed questions on consumption of poultry and other products (such as eggs and selected produce items) and purchase information such as date and location of purchase, shopper card numbers, and packaging information. State and local public health officials also attempted to identify localized illness sub-clusters, defined as events attended by 2 or more persons infected with 1 of the outbreak strains. Identified sub-clusters were further investigated to determine common food items consumed.

The proportion of case-patients reporting exposure to specific foods in the 7 days preceding illness onset was compared to the proportion reported in interviews of healthy persons in the FoodNet Population Survey [5], a population-based survey conducted during 2006–2007 in the United States that included questions about food consumed during the 7 days before interview. A binomial probability distribution was used to determine which food exposures reported by case-patients were significantly higher than those reported by healthy persons.

To assess the trajectory of the outbreak and to help determine when the outbreak ended, the number of reported illnesses was compared with the number that would be expected in the absence of an outbreak. This was calculated as the mean number of illnesses from the outbreak strains reported each week to PulseNet during 2010–2012, excluding illnesses associated with recognized illness clusters during that time period. Additionally, each of the 7 PFGE patterns was monitored with an outbreak detection algorithm based on CUSUM analysis [6], which statistically compares the number of illnesses in a given week to the seasonally adjusted 5-year mean number of cases and standard deviation. Data were analyzed with Microsoft Excel and SAS version 9.3 (SAS Institute, Cary, NC).

### Product traceback investigation

State and local health departments and USDA-FSIS conducted traceback investigations of chicken products consumed by case-patients. Product information, such as type of chicken product, brand, and date and location of purchase, was collected. Public health officials received permission to retrieve case-patient purchase information using shopper card numbers. USDA-FSIS assisted with traceback investigations from July 2013 through July 2014, focusing on information obtained from case-patient purchases identified through shopper card histories.

### Product testing

Several case-patients reported consuming chicken parts that were from multiple piece packages and freezing the unused raw pieces in the week before illness onset. Public health officials collected the leftover frozen raw chicken from case-patient homes. In addition, in September 2013, USDA-FSIS initiated intensified sampling of chicken products at 4 Company A chicken production establishments (3 in California, 1 in Washington) that were identified through traceback. After analysis of the initial sampling results, testing was expanded for a short period of time to 2 additional Company A establishments in Louisiana (where no samples yielded *Salmonella*) and continued at the 3 California establishments through October 2014. Leftover and in-facility chicken samples were cultured for *Salmonella*, serotyped, and subtyped by PFGE at state public health or USDA-FSIS laboratories.

### Antimicrobial susceptibility testing

CDC NARMS performed antimicrobial susceptibility testing (AST) on selected clinical isolates from humans and isolates from chicken samples from case-patients’ homes using standard NARMS methods [7]. FDA NARMS performed AST on *Salmonella* isolated through routine retail meat surveillance. All *Salmonella* isolates tested by NARMS and USDA-FSIS that were suspected of being part of this outbreak were serotyped and subtyped by PFGE; drug resistance was defined as resistance to 1 or more antimicrobials, and multi-drug resistance (MDR) was defined as resistance to at least 1 antimicrobial in 3 or more drug classes, as defined by the Clinical and Laboratory Standards Institute (CLSI).

## Results

### Case-finding and patient demographics

**Fig 1:**
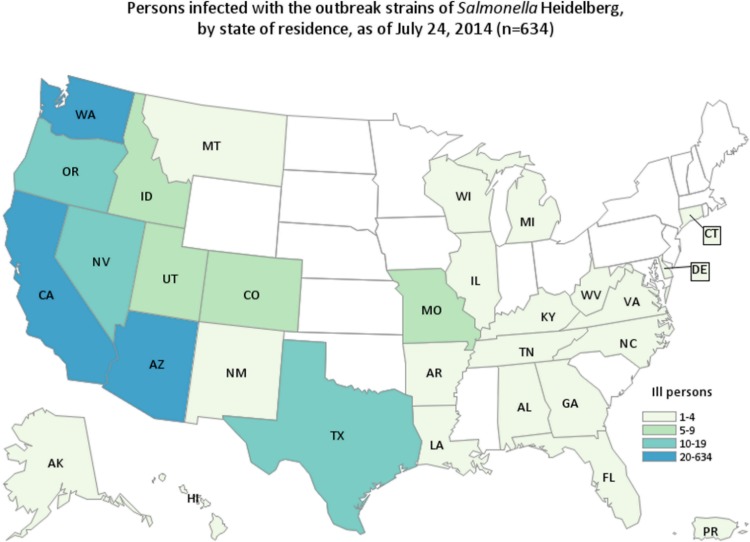
Persons Infected with the Outbreak Strains of *Salmonella* Heidelberg, by Week of Illness Onset, March 2013-July 2014.

**Fig 2:**
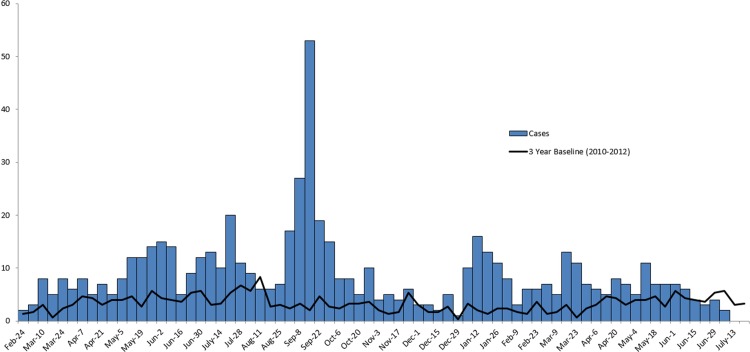
Persons Infected with the Outbreak Strains of *Salmonella* Heidelberg by State, n = 634, 2013–2014.

We identified 634 case-patients infected with 1 of the outbreak strains of *Salmonella* Heidelberg in 29 states and Puerto Rico with illness onset dates from March 1, 2013 through July 11, 2014 (Fig 1). Seventy-seven percent of the case-patients were reported from California (Fig 2). Case-patients ranged in age from less than 1 year to 93 years, with a median age of 18 years; 50% of case-patients were male (Table 1). Outbreak strains were isolated from blood specimens for 15% of case-patients. Among 528 case-patients with available information, 200 (38%) were hospitalized. No deaths were reported.

**Table 1:** Case-patient Characteristics in Multistate Outbreak of Multidrug-Resistant *Salmonella* Heidelberg Infections Linked to a Single Poultry Company, by Pulsed-Field Gel Electrophoresis Pattern, 2013–2014.

Characteristic	Total Outbreak		Pulsed-Field Gel Electrophoresis Pattern					
		JF6X01.0022^¥^	JF6X01.0041	JF6X01.0045	JF6X01.0122	JF6X01.0258	JF6X01.0326	JF6X01.0672
Total number case-patients	634	115	103	158	39	186	31	2
Reported and estimated onset date range	Mar 1, 2013 –Jul 7, 2014	Mar 1, 2013 –Jul 7, 2014	Mar 3, 2013 –Jun 25, 2014	Mar 11, 2013 –May 21, 2014	Mar 25, 2013 –May 17, 2014	Mar 9, 2013 –Jun 12, 2014	Mar 1, 2013 –Nov 2, 2013	May 2, 2013 –Jun 12, 2013
Median age (range) [years]	18(<1–93)	19(<1–93)	20(<1–90)	17(<1–91)	15(<1–88)	18(<1–87)	16(<1–85)	9(<1–18)
Male (%)	50%	47%	44%	47%	51%	57%	51%	50%
Hospitalized (%)	38%	38%	49%	42%	39%	31%	36%	50%
Frequency of PFGE pattern among *Salmonella* Heidelberg in PulseNet, 2013	N/A	44%	3%	2%	5%	<1%	3%	New pattern
Percent case-patients consuming any chicken in the week before becoming ill	86%	87%	83%	86%	73%	91%	95%	50%
Percent case-patients consuming Company A chicken in the week before becoming ill*	84%	76%	79%	76%	78%	95%	75%	100%

^¥^Only California case-patients qualified for inclusion

*Among those reporting chicken exposure who had brand information available

### Epidemiologic investigation

Interviews were conducted with 435 case-patients using initial or focused questionnaires: 371 (85%) reported eating chicken in the 7 days before becoming ill. Of 273 case-patients who completed the focused questionnaire, 201 (74%) reported eating chicken prepared at home (Table 1). This proportion was significantly higher than the FoodNet Population Survey, in which 65% of healthy persons interviewed reported eating chicken prepared at home in the 7 days before they were interviewed (p = 0.0004). Among 175 case-patients who had brand information available, 152 (87%) reported consuming Company A brand chicken or another brand likely produced by Company A. Among case patients who reported consuming chicken prepared at home and chicken type, 86% reported chicken parts (*e*.*g*., thighs, breasts, and wings) and 14% reported only whole chickens.

In October 2013, a common-source outbreak of *Salmonella* Heidelberg pattern JF6X01.0258 infections was linked to a single warehouse store location in California. A total of 32 case-patients were identified, including 9 illness sub-clusters of patients who shared meals at home or at gatherings. Case-patient store purchase histories confirmed that rotisserie chicken was the only common item purchased among ill persons. The rotisserie chickens were supplied raw by Company A to the store where they were cooked.

### Product traceback investigation

Eighty-two shopper card records from case-patients residing in Arizona, California, Michigan, Nevada, Utah, and Washington were used to trace chicken products to their source. Although USDA-FSIS traced raw chicken products to 6 Company A establishments, over 75% of the products were processed at the 3 Company A establishments in California. Traceback did not link the illnesses to a specific chicken product type, production period, or lot.

### Product testing

Nineteen leftover Company A raw chicken samples were collected from 13 case-patients’ homes; outbreak strains were isolated from 9 samples (Table 2). For 3 samples, the outbreak strain isolated from the case-patient was different from the outbreak strain isolated from the leftover chicken sample (Table 2).

**Table 2:** Leftover Chicken Products Collected from Case-Patient Homes, Multistate Outbreak of Multidrug-Resistant *Salmonella* Heidelberg Infections Linked to a Single Poultry Company, 2013–2014.

Chicken Sample	Month Tested	Case-patient Residency	Type of Product	Product PFGE Results	Patient PFGE Results	Product Information
Sample 1	August 2013	Washington	Unopened chicken parts	JF6X01.0672	JF6X01.0672	Packaging information insufficient to identify a specific lot/production period
Samples 2 and 3	October 2013	California	Rotisserie chicken (2 patients’ homes)	JF6X01.0258	JF6X01.0258	Purchased from California store; traced back to Company A, store recalled chicken products
Sample 4	November 2013	Michigan	Rotisserie chicken salad	JF6X01.0045	JF6X01.0045	Grocery store brand; chicken source unknown
Sample 5	October 2013	California	Company A boneless skinless chicken tenders	JF6X01.0122	JF6X01.0041	Purchased July or August 2013
Sample 6	February 2014	California	Company A boneless skinless chicken breasts	JF6X01.0258	JF6X01.0045	Purchased January 2014
Sample 7	March 2014	California	Company A boneless skinless chicken breasts	JF6X01.0041 and a *Salmonella* Hadar strain	JF6X01.0045	Purchased October or November 2013
Sample 8	April 2014	California	Company A fryer chicken thighs and drumsticks	JF6X01.0258	JF6X01.0258	Purchased March 2014; Packaging information insufficient to identify a specific lot/production period
Sample 9	July 2014	California	Company A boneless skinless chicken breasts	JF6X01.0258	JF6X01.0258	Recalled product included fresh and frozen chicken products produced in 3 California establishments from March 7 through March 13, 2014

During September 2013, USDA-FSIS testing in 3 Company A establishments in California identified *Salmonella* in 26% (116/450) of chicken samples, including 73 samples with 1 or more isolates that had PFGE patterns indistinguishable from 1 of the outbreak strains. Of 149 chicken samples collected at a Company A establishment in Washington, only 2 (1%) yielded *Salmonella*. Because of these findings, USDA-FSIS continued intensified sampling at the 3 California establishments. A total of 2,649 product samples were collected from the 3 California establishments, ultimately yielding 6 of 7 outbreak strains from raw whole chicken or parts. Four of the outbreak strains were identified across all 3 establishments. Over the subsequent months, a gradual decline in *Salmonella* incidence was seen in all 3 establishments. By October 2014, the percentage of chicken samples yielding *Salmonella* was approximately 5%.

### Antimicrobial susceptibility testing

CDC completed antimicrobial susceptibility testing of 69 clinical isolates, including all 7 outbreak strains. Of the 69 isolates tested, 22 (32%) were susceptible to all antimicrobials tested and 46 (67%) were drug resistant, including 24 (35%) that were MDR. One additional isolate was chloramphenicol intermediate, but was susceptible to all other antimicrobials tested (Table 3). Clinical isolates exhibited resistance to various combinations of ampicillin, chloramphenicol, gentamicin, kanamycin, streptomycin, sulfisoxazole, and tetracycline.

**Table 3:** Antimicrobial Susceptibility Testing Results, Multistate Outbreak of Multidrug-Resistant (MDR)^†^ *Salmonella* Heidelberg Infections Linked to a Single Poultry Company, 2013–2014.

Isolate Source	Total Outbreak	Pulsed-Field Gel Electrophoresis Pattern						
		JF6X01.0022^¥^	JF6X01.0041	JF6X01.0045	JF6X01.0122	JF6X01.0258	JF6X01.0326	JF6X01.0672
Case-patients	69 isolates tested: 46 (67%) resistant to ≥1 antimicrobial and 24 (35%) MDR	10 isolates tested: 1 resistant to aminoglycosides and sulfonamides, 3 MDR and 6 pansusceptible	13 isolates tested: 8 resistant to aminoglycosides and tetracyclines, and 5 MDR	14 isolates tested: 13 MDR, and 1 pansusceptible	8 isolates tested: All pansusceptible	14 isolates tested: 13 resistant to aminoglycosides and tetracyclines, and 1 MDR	8 isolates tested: 1 intermediate interpretation to chloramphenicol, and 7 pansusceptible	2 isolates tested: All MDR
Company A products collected from case-patients’ homes and store linked to sub-cluster	5 isolates tested: 4 (80%) resistant to ≥1 antimicrobial; 1 (20%) MDR	N/A	N/A	N/A	1 isolate tested: pansusceptible	3 isolates tested: All resistant to aminoglycosides and tetracyclines	N/A	1 isolate tested: MDR
Retail chicken samples	9 isolates tested: 9 (100%) resistant to ≥1 antimicrobial, and 3 (25%) MDR	1 isolate tested: resistant to aminoglycosides and tetracyclines	3 isolates tested: 2 resistant to aminoglycosides and tetracyclines, and 1 MDR	1 isolate tested: 1 MDR	1 isolate tested: 1 resistant to aminoglycosides and tetracyclines	3 isolates tested: 1 resistant to aminoglycosides and sulfonamides and 1 resistant to aminoglycosides and tetracyclines, and 1 MDR	N/A	N/A

^¥^Only California case-patients qualified for inclusion

†Defined as resistance to 1 or more agents in 3 or more antimicrobial classes

CDC also performed AST on *Salmonella* Heidelberg isolated from 5 Company A products; 4 were collected from case-patient homes (3 in California and 1 in Washington) and 1 from the California store associated with the illness sub-clusters. One isolate was susceptible to all antimicrobials and 4 (80%) were drug resistant, including 1 isolate that was MDR. These isolates were resistant to various combinations of kanamycin, streptomycin, sulfisoxazole, and tetracycline (Table 3).

FDA performed AST [8] on 9 *Salmonella* Heidelberg isolates from Company A chicken breasts and wings collected from retail stores in California (Table 3). These 9 isolates represented 5 of the outbreak strains. All 9 isolates were drug resistant, including 3 MDR isolates. These were resistant to various combinations of ampicillin, chloramphenicol, gentamicin, kanamycin, streptomycin, sulfisoxazole, and tetracycline.

### Control measures and resolution of the outbreak

On October 7, 2013, USDA-FSIS issued a public health alert due to concerns that *Salmonella* Heidelberg illnesses were associated with raw chicken products produced at the 3 Company A establishments in California. This alert reminded consumers of the critical importance of following cooking instructions and general food safety guidelines when handling and preparing any raw meat or poultry. On October 7, 2013, USDA-FSIS also issued a Notice of Intended Enforcement (NOIE) to the 3 Company A establishments in California. On October 8, 2013, CDC posted an outbreak investigation announcement on its website, informing the public of the investigation. On October 10, 2013, USDA-FSIS announced that Company A submitted and implemented immediate and substantive changes to its poultry production process. On October 12, 2013, the California store associated with the illness sub-clusters voluntarily recalled all cooked Company A rotisserie chicken products produced from September 11 through September 23, 2013 due to possible *Salmonella* contamination. On October 17, 2013, the recall was expanded to include additional products related to the rotisserie chicken produced from September 24, 2013 through October 15, 2013.

In October 2013, following the issuance of the NOIE, Company A began implementing measures in its poultry production and processing to decrease *Salmonella* burden for the 3 California establishments. According to the company, its strategy included interventions among breeder flocks, at hatcheries, at grow-out farms where chickens are raised for meat production, and at the chicken processing establishments. Company A introduced new *Salmonella* sampling programs during live production at grow-out farms and throughout processing. These programs included environmental monitoring of individual farms and assessing potential impact from neighboring farms. Based on the results of these programs, Company A developed environmental control procedures in and around the poultry houses to help reduce *Salmonella* transmission between flocks. Company A also made adjustments to operations, facility equipment, and employee training at its processing plants.

On July 3, 2014, Company A recalled an undetermined amount of chicken products that may have been contaminated with *Salmonella* after *Salmonella* Heidelberg PFGE pattern JF6X01.0258 was isolated from a raw leftover Company A chicken product collected from the home of a case-patient infected with the same strain. The recall included fresh and frozen chicken products produced in the 3 California establishments from March 7 through March 13, 2014.

By July 30, 2014, the weekly number of reported infections due to the outbreak strains returned to the expected seasonal baseline and the outbreak was declared to be over (Fig 1).

## Discussion

Epidemiologic, laboratory, traceback, and environmental investigations identified Company A chicken products from 3 production establishments in California as the source of the largest known multistate foodborne outbreak linked to consumption of chicken. Identifying the source of infections was challenging because chicken consumption is common, Company A has a large market share in the western United States, and the implicated products were sold under multiple brand names and produced in 3 different establishments. Obtaining detailed purchase information from case-patient interviews and grocery store purchase histories was essential to determine a link to Company A products. NARMS retail meat surveillance provided an important early clue on the source of the outbreaks and later identified multiple outbreak strains in Company A products with antimicrobial resistance patterns similar to those found in clinical isolates. This further supported a link between the illnesses and Company A chicken products. The epidemiological link to chicken consumption, traceback information leading to specific establishments where outbreak strains were identified, and numerous Company A chicken samples yielding the outbreak strains demonstrated that Company A chicken products were the source of this outbreak.

The high proportion of hospitalized case-patients (38%) and blood isolates (15%) documented in this outbreak is noteworthy [9]. Previous research has demonstrated an association between MDR strains of *Salmonella* and increased risk of hospitalization [2]. Antimicrobial resistance, including MDR, is an emergent problem with *Salmonella* Heidelberg [7, 10–12]. The AST findings in this investigation not only provide a potential explanation for high hospitalization rates among case-patients, but also suggest that selective pressure for antimicrobial-resistant strains exists in the environmental or animal reservoir (*e*.*g*., chicken litter, feed, pests, rodents). Of concern, in the last 3 years, at least 3 other multistate MDR *Salmonella* outbreaks have been linked to the consumption of poultry products [13–15]. Although clinical isolates were not resistant to antimicrobials typically used to treat severe *Salmonella* infections, these resistance patterns might be associated with more adverse outcomes. The recent frequency of MDR *Salmonella* outbreaks highlights the need to evaluate the impact of antimicrobial use and other management practices in the poultry industry on resistance in pathogens causing human disease such as *Salmonella*.

There have been several other recent poly-clonal salmonellosis outbreaks caused by either multiple serotypes or multiple PFGE patterns within the same serotype [16–19]. These outbreaks have been related to complex scenarios of contamination early in production or processing. Although subtype-based surveillance typically identifies a single PFGE pattern initially, poly-clonality is suspected when an increase occurs in several PFGE patterns with similar epidemiologic features, or when multiple patterns are isolated from a patient, an implicated food, or a production environment.

USDA-FSIS sampling showed that 6 of the outbreak strains were found among the 3 California production establishments. Further characterization demonstrated similar antimicrobial susceptibility patterns between Company A chicken isolates and case-patient isolates. These data suggest that the 3 production establishments likely shared a common source of contamination. This may be the result of an environmental reservoir at the farms or animal reservoir in the breeder or multiplier flocks that gives rise to broiler chickens that are eventually slaughtered and processed for human consumption. *Salmonella* Heidelberg is 1 of several *Salmonella* serotypes that is known to be passed transovarially from hen to progeny [20]. Comprehensive intervention strategies involving all phases of production from farm to processing plant to consumer are essential to prevent additional illnesses linked to poultry products.

An example of industry-wide pathogen control in meat production occurred in 2003, when USDA-FSIS and industry actions successfully reduced the frequency of *E*. *coli* O157:H7 in raw ground beef samples, likely leading to a subsequent decrease in human *E*. *coli* O157:H7 infections [21]. USDA-FSIS poultry performance standards instituted after the 1996 final rule on pathogen and hazard analysis and critical control points (HACCP) systems focused on reducing contamination of whole poultry carcasses at slaughter establishments [22]. A 2007–2008 Young Chicken Baseline Survey conducted by USDA-FSIS found that 5.9% of post-chill whole chicken carcasses yielded Salmonella [23]. Prior to the current outbreak, regulatory and industry attention focused on whole chicken carcasses rather than parts as there were no performance standards for parts. In this outbreak, 86% of case-patients reported consuming chicken parts versus only 14% who reported consuming whole chickens. Furthermore, baseline testing of chicken parts in 2012 by industry and USDA-FSIS found that an average of 26% yielded *Salmonella* [23]. In January 2015, USDA-FSIS proposed new performance standards to reduce *Salmonella* and *Campylobacter* in chicken parts and comminuted chicken and turkey products. The new standards [24] will allow 15.4% of chicken part samples at a processing facility to yield *Salmonella*.

There were limitations to this investigation. First, a formal analytic study was not conducted to statistically associate chicken consumption with illness. Case-patient food exposures were compared to those reported by healthy persons in the 2006–2007 FoodNet Population Survey. Poultry consumption may have changed since then and this comparison could have biased the association between chicken exposure and illness. However, according to the USDA Economic Research Service [25], chicken consumption peaked at 86.9 pounds per capita in 2006 and has remained virtually unchanged. Additionally, because case-patients are often interviewed about exposures several weeks after onset of illness, many have difficulty remembering specific foods or product and brand information. Finally, pattern JF6X01.0022 isolates from case-patients in states other than California that may have been related to the outbreak were excluded. Despite these limitations, the laboratory results from retail chicken samples, case-patient leftover chicken samples, and in-facility environmental and product testing provided strong evidence that Company A chicken products were the source of the outbreak.

The magnitude of this outbreak and subsequent investigation and actions by CDC, FSIS and industry investigation led to the implementation of important long-term changes in poultry processing and production practices. Specifically, Company A implemented multiple measures to decrease the *Salmonella* burden throughout its entire poultry production process at the 3 California establishments, reducing prevalence on chicken and chicken parts to approximately 5%. These measures may serve as model standards for other poultry producers and processors. The findings from this investigation highlight the importance of stringent food safety standards throughout the poultry industry. While consumer education and public messaging is important, the recent changes in chicken production and regulation will have a long-term impact on reducing the foodborne infections associated with poultry consumption and ultimately help reduce the foodborne salmonellosis incidence/burden. USDA-FSIS estimates that the new standards [24] for *Salmonella* and *Campylobacter* in chicken parts and comminuted poultry will prevent an estimated 50,000 illnesses each year. This large multistate outbreak illustrated the complexity of investigating poultry as a possible outbreak source, highlighted issues surrounding outbreaks of multidrug resistant *Salmonella*, and contributed to strengthening USDA Food Safety and Inspection Service (FSIS) food safety policies related to *Salmonella* in chicken parts.
